# Southern Medical Students

**Published:** 1850-11

**Authors:** 


THE MEDICAL EXAMINER.

PHILADELPHIA, NOVEMBER, 1850.

### SOUTHERN MEDICAL STUDENTS.

"We are sorry to find in a Southern religious paper the following article, so unjust to the North, and so prejudicial to that kindly feeling which has heretofore been perpetuated between the northern and southern colleges, and it is believed, to the advantage of both. The writer is eulogizing one of the southern medical colleges, earnestly and no doubt justly lauding its Faculty, which all seems proper and right. But is it necessary for the prosperity of any southern school, that so unfounded a statement should be put forth, as that which here alleges that 'in Philadelphia and New York, southern students are uniformly held in the utmost contempt,' and subjected to 'brutal treatment;' and hence urges upon such the duty of deserting northern schools?

There are scattered through the south many thousands of physicians, who were themselves professionally educated at the north, either in the schools of Philadelphia or New York, and whose pupils have annually been sent hither; upon whom the duty would seem to devolve, to repel these unheard of accusations, by testifying what was their reception and treatment from the 'Northerners' in these cities. It does seem to us that at the present crisis, when conciliation and compromise between citizens of our common country, is encouraging the hope of a better feeling both in the north and south, the introduction of so offensive an artible, in relation to our medical colleges, into any public journal, is singularly unfortunate.

On behalf of Philadelphia, as well as New York, we utterly deny that there is any foundation for the allegations made here against the Northerners; or for the prejudices here sought to be created against any of the medical colleges in this region. Nor can we believe that medical students from the south can be deterred from coming hither, by reason of such accusations made by an anonymous writer, without any semblance of evidence, or any attempt at proof.

We honour the laudable and successful efforts of our professional brethren in the southern section of our country, to found and sustain medical colleges, and other southern institutions of learning, so that they may no longer be dependent on the north, as they formerly were, to some extent and for obvious reasons. And when they shall be able to present facilities for the pursuit and cultivation of medical science, and clinical attractions to students, at any of these colleges, surpassing those so long existing at Philadelphia and New York, southern students will not be slow in estimating their claims, and awarding their preference to those teachers and schools which excel; and this without reference to their geographical position. Let our friends in Virginia make a greater and better medical school than we have here, and northern as well as southern students will flock thither. But until they do this, they should have higher reasons than 'abolition encroachments from the north and east,' to offer as inducements in favor of 'southern institutions;' and should not draw on their imagination for their facts by creating prejudices, as ungenerous as they are untrue, against their northern brethren.

'It is probable, that the reaction which must soon take place in favor of southern institutions generally, against the north and east, in repelling their abolition encroachments, will influence the prosperity of this excellent School. It is a matter of astonishment, that southern students should continue to throng northern medical schools, especially those of Philadelphia and New York, when they are *uniformly held by Northerners in the utmost contempt;* and when, too, they have experienced so many acts of unkindness, not to say *brutal treatment* at their hands. Southern students seldom ever receive their due measure of justice from the civil authorities of Philadelphia, should they chance to become embroiled with the citizens of that place. It is not unusual for a southern student, in such cases, to be held to bail, while the Philadelphians are exempted, and generally, too, when they are the aggressors. It is high time that southern students should resent such indignities, and this they can effectually do by deserting their schools, and patronizing southern institutions, which, generally, are little inferior to them in the means of instruction; and fully their equals, if not their superiors, in point of talent and ability of their professors.' "—*R. C. Advocate.*

We have extracted the above from the New York Medical Gazette, to the Editor of which our thanks are due for his defence of Philadelphia. We are sure that all he says of New York is true, and we *know* that in this city, students from all sections are uniformly treated with kindness by all. That they do occasionally become embroiled with the citizens is doubtless true; but that they receive full justice, and even indulgence from the anthorities, the following letter from the Attorney General ot the Commonwealth will show.

*Philadelphia, Oct.* 22*d*, 1850.

Dear Sir,

There is not the slightest foundation for the idea that Southern medical students are, or ever have been, unjustly, or even unkindly, treated by the authorities of Philadelphia. For more than two years have conducted the public business, as prosecuting officer for this City and County, and have of course had full opportunities of observation. Nothing of the kind has occurred; so far from it, that in a few instances within my recent recollection, there has been a strong disposition to look mercifully on outbreaks of irregular and excited young men. The Professors in our colleges here, many of them being Southern gentlemen by birth and early association, have always been ready to enter bail for those young gentlemen who get themselves into trouble, and discredit their respectable families at a distance; and here the matter usually drops. I am happy to say that, within the last few years, there has been a great improvement in the demeanor of medical students from abroad. They are generally very respectable and decorous young men, are cordially welcomed, and kindly and justly treated when they come and whilst they stay amongst us.

I hope you will peremptorily contradict this absurd story.

Truly yours, WILLIAM B. REED.

DR. F. G. SMITH.

We have often said with pride, of medical students, that we did not believe that an equal number of young men of any other profession could be assembled, against whom so few causes of complaint could be urged. We do them no more than justice in bearing this witness, and to them we look for the refutation of the charges that have been so ungenerously urged against New York and Philadelphia.

